# Editorial: Bioengineered Nanoparticles in Cancer Therapy

**DOI:** 10.3389/fmolb.2021.706277

**Published:** 2021-07-13

**Authors:** Payam Zarrintaj, Masoud Mozafari, Henri Vahabi, Tomy J. Gutiérrez, Mohammad Reza Saeb

**Affiliations:** ^1^ Department of Biomedical and Pharmaceutical Sciences, University of Montana, Missoula, MT, United States; ^2^ Department of Tissue Engineering and Regenerative Medicine, Faculty of Advanced Technologies in Medicine, Iran University of Medical Sciences, Tehran, Iran; ^3^ Université de Lorraine, CentraleSupélec, LMOPS, Metz, France; ^4^ Grupo de Materiales Compuestos Termoplásticos (CoMP), Instituto de Investigaciones en Ciencia y Tecnología de Materiales (INTEMA), Facultad de Ingeniería, Universidad Nacional de Mar del Plata (UNMdP) y Consejo Nacional de Investigaciones Científicas y Técnicas (CONICET), Mar del Plata, Argentina; ^5^ Department of Resin and Additives, Institute for Color Science and Technology, Tehran, Iran

**Keywords:** bioengineering, biomaterials, cancer therapy, nanoparticles, nanotechnology

Cancer is a genetic disease associated with the rapid growth of abnormal cells, which provoked the death of almost 10 million people worldwide during 2020 (WHO, 2021a), placing it among the top 10 causes of death worldwide (WHO, 2021b). In this context, numerous efforts have been made with the aim of preventing, detecting and treating this disease effectively without major side effects in terms of damage to healthy tissues, insulin resistance, Orthostatic hypotension, among others. In particular, engineered nanoparticles are currently playing an important role in this field as controlled release systems for anticancer drugs (Shi et al., 2017; Khodadadi Yazdi et al., 2020), theragnostic devices (Chauhan and Jain, 2013; Gutiérrez and Alvarez, 2018; Gutiérrez, 2018; Wolfram and Ferrari, 2019, among others.

Traditional cancer treatments have shown several limitations. Nonetheless, diverse technologies based on nanotechnology have shown significant advances with the aim of obtaining a more efficient and safe cancer therapy. Despite this, several key obstacles related to the use of nanoparticles for cancer therapy such as the complexity and heterogeneity of tumor biology, a lack of understanding of nano-bio interactions, as well as chemical, manufacturing and control challenges must be further studied for clinical success. This research topic addresses some novel aspects of engineering that take advantage of our growing understanding of bionano behaviors and interactions to develop more efficient nanotherapies for cancer patients. Keeping this in mind, in this research topic, Sanità et al. reviewed the most recent techniques for surface modification and functionalization of nanoparticles in order to improve their biocompatibility and cellular uptake behavior. Similarly, Cheng et al. summarized and analyzed the current research progresses and challenges in tumor microenvironment-responsive shrink-sized drug delivery nanosystems. Cheng et al. also discussed the current implications and knowledge for promoting deep penetration into tumors using nanoparticles. Meanwhile, Chen et al. used two novel HLA-A2-restricted cytotoxic T lymphocyte epitopes (SV_95–6_ and SV_95–7_ peptides) derived from survivin (SV, pectic tumor antigen), and were then loaded into human dendritic cell/poly(lactic-*co*-glycolic) acid-based nanoparticles, thus obtaining advanced materials specific against cancer cells. It should also be noted that Chen et al. carried out major histocompatibility complex peptide binding algorithms to predict a range of modified SV_95_ decamers (from SV_95–2_ to SV_95–10_) based on the natural SV_95–104_ peptide sequence of ELTLGEFLKL. On the other hand, Sharifiaghdam et al. designed and synthesized new layer-by-layer selenium-based nanocomplexes as carriers of small interfering RNA with improved stability and a dual mode of action against tumors: gene silencing and apoptosis induction in cancer cells. To close this research topic, Shah et al. reported theranostic optical imaging probes based on shortwave infrared (SWIR)-emitting rare earth-doped nanoparticles encapsulated with human serum albumin (ReANCs), which demonstrated superior surveillance ability for detecting micro-lesions at depths of 1 cm in an animal models of breast cancer metastasis, thereby promising an ability for follow-up therapy based on SWIR fluorescence measurements from tumor-targeted ReANCs.

The role of nanotechnology in cancer research has grown dramatically in recent years. However, only a few dozen nano-based technologies have reached the market so far, primarily cell-scale targeted bionanosystems, and controlled and sustained carries of desired biomolecules. To change this, we must reconsider traditional views and rethink how we conduct translational cancer nanomedicine research.
